# Association Between *Chlamydia trachomatis* and *Helicobacter pylori* with Inflammation in Polycystic Ovary Syndrome: A Cross-Sectional Study

**DOI:** 10.3390/medicina60122102

**Published:** 2024-12-22

**Authors:** Yeşim Alpay Çağlar, Mine Islimye Taşkin

**Affiliations:** 1 Department of Infectious Disease and Clinical Microbiology, Balıkesir University School of Medicine, 10145 Balıkesir, Turkey; 2 Department of Obstetrics and Gynecology, Balıkesir University School of Medicine, 10145 Balıkesir, Turkey

**Keywords:** polycystic ovary syndrome, inflammation, infection, *Helicobacter pylori*, *Chlamydia trachomatis*

## Abstract

*Objective*: Chronic low-grade inflammation occurs in polycystic ovary syndrome (PCOS), and there are many contributing factors. In this study, we aimed to investigate *Helicobacter pylori* and *Chlamydia trachomatis* infections in patients with PCOS and to evaluate the association between these microorganisms and the inflammatory process in the etiology of the disease. *Materials and Methods*: This comparative cross-sectional clinical study was conducted at Balıkesir University Hospital and included 40 female patients diagnosed with PCOS in the gynecology outpatients clinic and 40 healthy female controls. Demographic data were recorded. Blood hormone profiles and biochemical parameters were analyzed. An enzyme-linked immunosorbent assay test kit was used to measure *H. pylori* IgG and *C. trachomatis* IgG. *Results*: According to the analysis of the study data, there was no significant association between the PCOS and non-PCOS groups with regard to the presence of *Helicobacter pylori* IgG (*p* = 0.1) and *Chlamydia trachomatis* IgG (*p* = 0.338). CRP levels were significantly higher in the PCOS group (*p* = 0.001). In the subgroup analyses, the CRP levels were not significantly different between the *H. pylori* and *C. trachomatis* antibody-positive and -negative groups. Diabetes mellitus was significantly associated with PCOS (*p* = 0.005). The smoking rate was significantly higher in the control group than in the PCOS group (*p* = 0.036). Compared to the control group, the BMI, LH, HOMA-IR, TSH, and TG levels were significantly higher in participants with PCOS (*p* = 0.000; *p* = 0.004; *p* = 0.001; *p* = 0.001; *p* = 0.043; *p* = 0.000). FSH was lower in PCOS patients compared to controls (*p* = 001). In the subgroup analyses, no significant differences were found between the *H. pylori* and *C. trachomatis* antibody-positive and -negative groups. *Conclusions*: PCOS is characterized by chronic nonspecific low-grade inflammation. The etiopathogenesis of PCOS involves comorbidities that cause a chronic inflammatory process. However, the possible infective causes still seem to be open to investigation. In particular, studies on microbiota and periodontal diseases in PCOS may provide important contributions.

## 1. Introduction

Polycystic ovary syndrome (PCOS) is a common cause of hormonal dysregulation affecting 8–13% of women of reproductive age. PCOS is a complex disease with hormonal, metabolic, and cardiovascular components such as coronary artery disease and long-term risks such as diabetes mellitus. Several factors play a role in its etiology. Increased visceral adipose tissue is associated with the comorbidities of hyperglycemia, dyslipidemia, and insulin resistance [1].

Chronic low-grade inflammation and oxidative stress play a role in the pathogenesis of PCOS [2]. Increased proinflammatory factors may cause systemic inflammation [3,4].

Polycystic ovary syndrome is associated with a marked increase in inflammation biomarkers such as C-reactive protein (CRP), IL-18, and MCP-1, and is also associated with other inflammation-related disorders [5]. Studies have shown that systemic inflammation is increased in patients with PCOS, and CRP may indicate this. The focus of the majority of studies addressing the status of chronic low-grade inflammation in patients with PCOS has been on CRP measurement. CRP is an acute-phase protein synthesized by the liver. CRP is also produced directly by adipose tissue.

Studies show that CRP is positively correlated with insulin resistance and body fat mass [6,7].

PCOS itself causes metabolic dysfunction such as dyslipidemia, endothelial dysfunction, atherosclerosis, insulin resistance, obesity, fatty liver disease, coagulation disorders, and an increased risk of cardiovascular disease [8]. There are also studies describing the interactive effect of hyperinsulinemia, obesity, hyperandrogenism, and inflammatory status [9,10].

The impact of infections on the presence of chronic inflammation in women with PCOS has occasionally been investigated [5]. Studies have been carried out to identify factors such as the presence of certain pathogens associated with this low-grade chronic inflammation in PCOS. Although there is a lack of studies in the literature investigating the relationship between PCOS and pathogens, it is thought that there is a relationship between PCOS and some infections [11,12,13].

Since *Chlamydia* spp. and *Helicobacter pylori* infections have been suggested to be associated with some chronic inflammatory diseases, it has been thought that they may also be associated with inflammation in PCOS [11]. However, this has not yet been clarified.

*Chlamydia* spp. and *Helicobacter pylori* infections are very common worldwide. Studies on these two pathogens have been of interest. In this study, *Chlamydia* spp. and *Helicobacter pylori* infections were selected because of their widespread prevalence in the community, their silent course of infection, and their strong influence on the development of chronic inflammation.

*Chlamydia trachomatis* infection is particularly common and is known to be strongly associated with infertility. It is an obligate intracellular pathogen with a slow growth cycle, and tends to be chronic and silent [7]. Case–control studies have suggested that *Chlamydia trachomatis* antibodies may contribute to pathogenetic processes that lead to chronic inflammation, cardiovascular diseases, and metabolic and hormonal disorders associated with PCOS [12,14,15]. In particular, *Chlamydia pneumonia* seropositivity has been associated with atherosclerosis and acute myocardial infarction [16]. Similarly, *H. pylori* is associated with local, gastric, and chronic systemic inflammation. Coronary heart disease, stroke, aneurysm, migraine, idiopathic arrhythmia, and endocrine system disorders are among the most common non-digestive system diseases and syndromes associated with *Helicobacter pylori* infection [13,17]. Because of the information on the association of these two pathogens with chronic inflammation, their role in the etiology of some chronic diseases has been investigated.

In this study, we aimed to investigate *Helicobacter pylori* and *Chlamydia trachomatis* infections in patients with polycystic ovary syndrome and to evaluate the association between these microorganisms and the inflammatory process in the etiology of the disease.

## 2. Materials and Methods

### 2.1. Participants and Study Design

In this comparative cross-sectional clinical study, conducted at Balıkesir University Faculty of Medicine, 40 female patients between the ages of 15 and 35 diagnosed with polycystic ovary syndrome in the gynecology outpatient clinic and 40 healthy female controls were included.

Ethical approval was obtained in accordance with the Declaration of Helsinki and was approved by the Ethics Committee of Balıkesir University Faculty of Medicine (number: 2017/50, date: 05/07/2017).

Diagnosis was defined according to the 2003 Rotterdam PCOS diagnostic criteria. Women with at least two of the following criteria were included in the PCOS group: (1) oligo- or anovulation; (2) clinical and/or biochemical hyperandrogenism; (3) polycystic morphology of the ovary on ultrasound examination (at least 12 follicles 2–9 mm in diameter and/or ≥10 mL volume per ovary) [18].

The diagnosis of oligo-anovulation was based on the presence of oligomenorrhea (a menstrual cycle longer than 35 days) or amenorrhea (the absence of menstruation for 6 months or longer). Blood samples for hormonal evaluation were collected during the early follicular phase of the menstrual cycle, and a transvaginal ultrasound was performed on the same day using a 7.5 MHz vaginal transducer (Voluson 730, GE Healthcare, Tiefenbach, Austria). Patients were excluded from receiving any treatment including insulin sensitizers, glucocorticoids, anticoagulants, antiandrogens, oral contraceptives, and antiplatelet drugs. Other causes of hyperandrogenism such as hyperprolactinemia, hypothyroidism, congenital adrenal hyperplasia, Cushing’s syndrome, and virilizing tumors were excluded for the diagnosis of PCOS. The Ferriman–Gallwey (FG) scale was used for the diagnosis of clinical hirsutism and a score higher than 8 was accepted as clinical hyperandrogenism [19]. For the diagnosis of biochemical hyperandrogenism, serum testosterone levels were measured by an ELISA using commercially available kits (eBioscience, Vienna, Austria) in a diagnostic device (BioTek, ELx 800, Winooski, VT, USA) [19].

The control subjects were healthy volunteers from the community without clinical menstrual disorders and with clinical evidence of hyperandrogenism. Women with any of the following conditions were excluded from the study: pregnancy or breastfeeding, diabetes mellitus, or acute infection within the last 3 weeks. Patients of advanced age; receiving anti-inflammatory, antimicrobial, corticosteroid, immunosuppression, or autoimmune therapy; known to have chronic disease (liver, kidney, autoimmune, neoplasia, Human Immunodeficiency Virus); or who had very high CRP levels or other known hyperandrogenemia conditions (late-onset congenital adrenal hyperplasia, Cushing’s syndrome, and androgenizing tumors) were excluded.

Patients’ presenting complaints, age, comorbidities, laboratory results, body mass index (BMI), and ultrasonographic findings were recorded. Body mass index (BMI) was calculated by dividing a person’s weight in kilograms by the square of their height in meters. The overweight limit was accepted as 25 kg/m^2^ and above. Blood hormone profiles including follicle-stimulating hormone (FSH), luteinizing hormone (LH), thyroid-stimulating hormone (TSH) levels, and biochemical parameters including low-density lipoprotein (LDL), high-density lipoprotein (HDL), triglycerides, cholesterol, fasting blood glucose (FBS), and CRP values were analyzed.

### 2.2. Biochemical Analysis

Venous blood samples taken from all patients between 09:00 and 10:00 after fasting overnight were sent to the laboratory for hormonal and biochemical analyses. All samples were centrifuged at 825× *g* for 10 min and stored at −40 °C until analysis. Commercially available kits (Cobas Integra 800; Roche Diagnostics GmbH, Mannheim, Germany) were used in a chemistry autoanalyzer to measure glucose, LDL, HDL, total cholesterol, and triglycerides. In a hormone autoanalyzer, we measured fasting insulin levels with Access Kits (Beckman Coulter; Unicel DXI 600; Access Immunoassay, Brea, CA, USA). CRP concentration was determined by laser nephrometry.

Insulin resistance was calculated using the homeostasis model [homeostasis model assessment for insulin resistance (HOMA-IR)], i.e., fasting glucose (mmol/L) × fasting insulin (mU/L)/22.5 [20].

All blood samples were sent to the same laboratory. A tube of blood was taken from the PCOS and control groups and analyzed in a biochemistry tube for *Helicobacter pylori* IgG and *Chlamydia trachomatis* IgG. All samples were centrifuged at 1300× *g* for 10 min and stored at −70 °C until analysis.

Enzyme-linked immunosorbent assay test kits were used to measure *Helicobacter pylori* IgG and *Chlamydia trachomatis* IgG (ELISA) (NovaTec, Immundiagnostica GmbH, NovaLisa^®^, Dietzenbach, Germany). In the method, approximately 10 µL of the participant’s serum was taken and diluted; each well was incubated at 37 °C for one hour and then washed with washing solution. After the conjugate was added, the plates were washed again after incubation at room temperature for another 30 min and TMB substrate was added. The samples were then incubated at room temperature in the dark for 15 min. Finally, stop solution was added to all wells in the same order and at the same rate as for the TMB. Samples with values 10% higher than the cut-off value were classed as positive and those with values 10% lower than the cut-off value were negative. The results for *Helicobacter pylori* IgG were interpreted as reactive if the absorbance value was >20 NTU/mL, non-reactive if the value was <15 NTU/mL, and in the gray zone if the value was between 15 and 20 NTU/mL. For the *Chlamydia trachomatis* IgG, values above 11 NTU (NovaTec units) were considered positive and those below 9 NTU were considered negative. Borderline samples with 9–11 NTU were classed as being in the gray zone.

### 2.3. Data Analysis

SPSS statistical software (SPSS, version 20.0, Chicago, IL, USA) was used for all statistical analyses. The following descriptive statistics were used to describe the study data: mean, median, standard deviation, and frequency. A comparison between the groups was made with the Student’s *t*-test. Correlation between data: those with no normal distribution (non-parametric) were evaluated with Spearman’s rho and the Mann–Whitney U test, and those with normal distribution (parametric) were evaluated with Pearson’s correlation. A *p*-value < 0.05 was statistically accepted.

## 3. Results

Forty patients with PCOS with a mean age of 27 (range, 21 to 38) years were included in the study. The control group consisted of 40 healthy individuals with a mean age of 27.5 (range, 19 to 35) years. Among comorbid diseases, diabetes mellitus (DM) and BMI were significantly associated with PCOS (*p* = 0.005, *p* = 0.000). The rate of smoking was significantly higher in the control group than in the PCOS group (*p* = 0.036). The distribution of the descriptive features and operating parameters is listed in Table 1.

*C. Trachomatis* IgG seropositivity was found to be 10% (4) in the PCOS group and 5% (2) in the control group. *H. pylori* IgG seropositivity was found to be 70% (28) in the PCOS group and 50% (12) in the control group. There was no significant association between the PCOS and control groups in terms of the presence of *H. pylori* IgG (*p* = 0.1) and *C. trachomatis* IgG (*p* = 0.338). The data are listed in Table 2.

Compared to the control group, the BMI, LH, HOMA-IR, TSH, and TG levels were significantly higher among the participants with PCOS (*p* = 0.000; *p* = 0.004; *p* = 0.004; *p* = 0.001; *p* = 0.043; *p* = 0.000). FSH was lower in PCOS patients compared to the controls (*p* = 0.001). The metabolic and hormonal features of the study groups are listed in Table 3.

The PCOS and control groups were subdivided into positive and negative groups for *C. trachomatis* and *H. pylori* antibody titers. Differences in metabolic complications and CRP levels were investigated in these subgroups. No significant difference was observed in the CRP levels in the *C. trachomatis* IgG- (*p* = 0.338) and *H. pylori* IgG (*p* = 0.1)-positive groups compared to negative groups (*p* = 0.419; *p* = 0.122). Among the metabolic parameters, the cholesterol and triglyceride values were significantly higher in the *C. trachomatis* IgG-negative patients (*p* = 0.008; *p* = 0.041). No significant difference was found for any of the other parameters (*p* > 0.05). A similar grouping was made in the control group, but no significant difference was observed between the antibody-negative and -positive groups in terms of CRP and metabolic parameters. The metabolic and hormonal features of the study subgroups are presented in Table 4.

## 4. Discussion

It has been suggested that chronic systemic inflammation plays a role in the development of PCOS. PCOS, which is characterized by hyperandrogenemia and chronic oligo/anovulation, is also associated with metabolic disorders. Some researchers have argued that PCOS represents the female subtype of metabolic syndrome with a potential atherogenic risk [21]. Increased markers such as CRP and leucocytes and the detection of inflammatory cytokines such as interleukin-6 and interleukin-18 in patients with PCOS indicate a low level of chronic inflammation [22,23,24,25,26].

It was predicted that useful information would be obtained from research on the infectious/inflammatory component of this chronic and multifactorial disease. Studies have been conducted to identify the factors associated with this low-grade chronic inflammation in PCOS, such as the presence of specific pathogens. It has also been hypothesized that this syndrome may be caused by infectious agents, although the exact pathogenic mechanisms are still unclear.

Studies have suggested a relationship between the presence of low-grade chronic inflammation and *Chlamydia* spp. or *Helicobacter pylori* [11,12,13]. However, there are a limited number of studies investigating the relationship with PCOS and it has not been sufficiently elucidated. In particular, although the study by Morin-Papunen et al. [12] on the association between chlamydial infections (*Chlamydia trachomatis* and *Chlamydia pneumoniae*) and PCOS has raised significant interest, no supporting study has been conducted and there are not many studies in the literature on this subject. In our study, we also aimed to address this issue.

In our study, *Chlamydia trachomatis* antibody positivity was found in 10% (4) of 40 women with PCOS. In the group of 40 healthy women, this rate was found to be 5% (2). No significant difference was found between the groups (*p* = 0.338).

In previous studies, *Chlamydia pneumoniae* seropositivity has been reported to be associated with myocardial infarction and atherosclerosis [11,16]. The significant association between *C. pneumoniae* seropositivity and higher BMI and metabolic syndrome also suggests that CRP plays an active role in the development of insulin resistance. Similarly, an association between *Helicobacter pylori* seropositivity and arterial stiffness and unstable angina has been reported [27,28].

*Helicobacter pylori* has been shown to cause both local gastric and chronic systemic inflammation. In the study conducted by Yavasoglu et al. [17], although *H. pylori* antibody positivity was found in 40% of women with PCOS, this rate was 22% in the healthy group, and *H. pylori* was associated with PCOS. In another study, no difference was found for *H. pylori* IgG seroprevalence between the PCOS and non-PCOS groups (*p* = 0.924) [29]. In our study, 70% of 40 women with PCOS were positive for *H. pylori* antibodies, whereas this rate was 50% in a healthy group of 40 women. No significant difference was found between the groups (*p* = 0.110).

In a different study, the relationship between latent *Cytomegalovirus* (CMV) and *Epstein–Barr virus* (EBV) infection and PCOS was investigated using anti-CMV and anti-EBV IgG-specific antibodies in serum samples. In 60 women with PCOS, 16.7% were found to be positive for CMV and only 1 case (3.3%) was positive in the control group. On the other hand, in the group with PCOS, 27 (45%) individuals were positive for anti-EBV IgG Ab, whereas none of the 30 women (0%) in the control group tested positive. Based on these results, it has been suggested that these viruses may be involved in one aspect of the causal events behind PCOS [30]. No other study has been conducted to support this.

Periodontal infections have been suggested to be related to the development of inflammation and chronic processes [13,31]. The pathophysiological relationship of systemic inflammation between PCOS and periodontitis is supported by studies. It is particularly interesting to note the high prevalence of periodontal disease in non-obese women with PCOS compared to healthy controls. In chronic infections such as periodontitis, it has been suggested that elevated serum levels of CRP and other proinflammatories may induce systemic inflammation and oxidative stress, which contribute to the insulin resistance observed in PCOS [32].

Insulin resistance is defined as the failure of a normal biological response to endogenous and exogenous insulin. Insulin resistance has been detected in 43–76% of people with PCOS. In these women, an increase in the frequency of Type 2 DM and 5–30% glucose intolerance have been found [33]. In our study, DM was the most common comorbidity in the PCOS patient group (*p* = 0.005). In women with PCOS, dyslipidemia and dysfibrinogenemia may develop due to hyperandrogenism and obesity, and the risk of coronary artery disease increases. Insulin resistance also contributes to dyslipidemia. The HDL/LDL ratio decreases and triglycerides increase. In studies, it has been found that lipid levels are borderline high in approximately 70% of women with PCOS [34,35]. Our study also supports this: BMI, HOMA-IR, and TG (*p* = 0.000, *p* = 0.001, *p* = 0.000) were significantly higher in subjects with PCOS compared with those of the control group. The mean CRP levels were 9.45 [2,3,4,5,6,7,8,9,10,11,12,13,14,15,16,17,18,19,20,21,22,23,24,25,26,27,28,29,30,31,32,33,34,35,36,37,38,39,40] in patients with PCOS and 3.45 [3,4,5,6,7,8] in the healthy group. It was found to be significantly higher in the PCOS group and was found to be consistent as a marker of inflammation.

In our study, we also investigated the differences in metabolic complications and CRP levels in subgroups with positive and negative *C. trachomatis* and *H. pylori* antibody titers in PCOS and healthy controls. No significant difference was observed in the CRP levels in *C. trachomatis* IgG- (*p* = 0.338) and *H. pylori* IgG (*p* = 0.1)-positive groups compared to negative groups (*p* = 0.419; *p* = 0.122). Among the metabolic parameters, the cholesterol and triglyceride values were significantly higher in *C. trachomatis* IgG-negative patients (*p* = 0.008; *p* = 0.041). No significant difference was found in any of the other parameters (*p* > 0.05). A similar comparison was made in the control group, but there was no significant difference between the antibody-negative and -positive groups in terms of CRP or metabolic parameters.

In the study of Kanber et al. [36], CRP, sICAM-1 and sE-selectin levels, which are markers of chronic inflammation, were found to be significantly higher in patients with PCOS than in the control group. Elevated CRP levels are observed in many systemic diseases, including PCOS pathogenesis, which is associated with low-grade chronic inflammation and hyperinsulinemia [22,37]. A meta-analysis of 31 clinical trials concluded that CRP was, on average, 96% higher in women with PCOS than in control groups [38].

In the results of our study, no significant difference was observed between the PCOS patients and healthy controls in terms of either microorganism. The CRP levels were significantly higher in the PCOS group. In the subgroup analyses, the CRP levels were not significantly different between the antibody-positive and -negative groups. These results are remarkable in terms of the non-infectious causes of chronic inflammation accompanying PCOS.

In Rudnizka et al.’s [39] study, the predictive factors of elevated CRP were mainly BMI and insulin resistance, but it was emphasized that inflammation may be associated not only with adiposity, but also with increased androgen concentration. In our study, high BMI, HOMA-IR, and DM values, which are predicted for elevated CRP, were present in the patient group with PCOS.

Many factors contribute to chronic low-grade inflammation in the pathogenesis of PCOS. For instance, abdominal obesity has been shown to cause metabolic and hormonal disruption in PCOS and visceral obesity has been shown to cause chronic low-grade inflammation [40,41]. Adipocytes secrete proinflammatory cytokines that facilitate the chronic inflammatory response. In our study, the BMI was significantly higher in the PCOS group, and metabolic conditions associated with high HOMA-IR and triglyceride levels were thought to contribute to inflammation. The contribution of metabolic conditions associated with PCOS to inflammation was considered.

Adipose tissue secretes a variety of signaling molecules that regulate specific homeostatic systems such as food intake, energy expenditure, insulin secretion, and insulin function. Most of these factors are cytokines such as tumor necrosis factor (TNF) alpha, CRP, interleukin (IL)-6, which regulate the immune response [42]. It has been reported that the expression profile of adipose tissue is altered in patients with PCOS, which leads to an imbalance in adipokine secretion, increased inflammation, impaired endothelial function, and insulin resistance, and negatively affects endocrine and reproductive systems [43,44].

In the course of PCOS, chronic inflammation is observed. The relationship with infections is controversial and the evidence is not strong enough to form an opinion. Although metabolic complications in PCOS explain inflammation without any association with infection, investigating the relationship with periodontal infections and microbiota may lead to important results. The “dysbiosis theory of gut microbiota”, which proposes increased intestinal mucosal permeability and increased lipopolysaccharide transit from Gram-negative intestinal bacteria, has been associated with PCOS. Permeability, immune system activation, insulin receptor dysfunction, insulin elevation, increased androgen production, and impaired follicle development may explain components of PCOS diagnostic criteria. It may be of current interest for microbial etiology [45].

In this study, we investigated the contribution of some infectious agents and discussed inflammation in the complex etiopathogenesis of PCOS. While emphasizing the non-infectious etiology, we also highlighted areas of research that we still consider important.

A limitation of this study is our relatively small sample size, which is not necessarily sufficient to produce conclusive outcomes. It would be desirable to repeat the study using a large, multicenter cohort. Studies investigating the relationship between PCOS and gut microbiota and periodontitis may make important contributions.

## 5. Conclusions

The relationship between inflammation and infections observed in the course of PCOS is controversial and unclear. In recent years, studies have been published showing a link between inflammation in PCOS and infectious agents such as *Chlamydia* spp. and *Helicobacter pylori*. However, there have also been studies that do not support this. Our study aimed to investigate the relationship between infection and inflammatory processes by investigating serum *Chlamydia trachomatis* and *Helicobacter pylori* antibodies in patients with PCOS and in healthy controls. There was no significant association between the PCOS and non-PCOS groups in terms of the presence of *H. pylori* and *C. trachomatis* antibodies. CRP levels were found to be significantly higher in the PCOS group and were not significantly different in the antibody-positive patients compared to the antibody-negative patients. This led us to discuss the factors that contribute to the chronic inflammatory process in the etiopathogenesis of PCOS. Increased visceral adipose tissue, insulin resistance, impaired glucose tolerance, and abnormalities in lipid metabolism are associated with PCOS. In our study, the DM, BMI, and HOMA-IR values were significantly higher in the patient group, supporting this. On the other hand, in the presence of current knowledge, it may be useful to keep investigating possible infectious causes. The role of inflammation and infections in the etiology of multifactorial diseases is always open to investigation. For PCOS, the theorem regarding the relationship between intestinal microbiota dysbiosis and PCOS in infectious etiology seems to be of interest. Studies on microbiota and periodontal diseases in PCOS patients may provide important contributions.

## Figures and Tables

**Table 1:** Distribution of descriptive features.

		PCOS		*p-Value*
		*Positive (n* = 40)	*Negative (n* = 40)	
**Age** (year)	***Min–Max (Median)***	21–38 (27)	19–35 (27.5)	0.919
	***Mean ± SD***	27.52 ± 4.14	27.42 ± 4.53	
**Comorbidity**	***None***	25	40	
	***Diabetes mellitus (DM)***	8	0	0.005 *
	***Hypertension (HT)***	1	0	
	***Thyroid disease***	4	0	
	***FMF***	1	0	
	***Anemia***	1	0	
**Marital status**	***Married***	33	25	0.127
	***Single***	7	15	
**BMI** (kg/m^2^)	***18.5–24.9***	9	40	
	***25–29.9***	15	0	0.000 *
	***30≤***	16	0	
**Smoking**	***Yes***	5	14	0.036 *
	***No***	35	26	

BMI, body mass index. * Values in bold in the *p*-value column are those found to be statistically significant. *p*-value < 0.05 was considered significant.

**Table 2:** *H. pylori* IgG and *C. trachomatis* IgG seroprevalence in PCOS and non-PCOS groups.

		PCOS		*p-Value*
		*Positive (n* = 40)	*Negative (n* = 40)	
***H. pylori* IgG**	***Positive***	28	20	0.110
	***Negative***	12	20	
***C. trachomatis* IgG**	***Positive***	4	2	0.338
	***Negative***	36	38	

*p*-value < 0.05 was considered significant.

**Table 3:** The metabolic and hormonal features of the study groups.

Variables	PCOS (*n* = 40)	Control (*n* = 40)	*p-Value*
**FSH** (IU/L)	6.2 (2.27)	8.02 (2.77)	**0.001** **
**LH** (IU/L)	8.47 (5.87)	5 (2.44)	0.004 *
**E2** (ng/L)	49.42 (54.10)	50.65 (43.45)	0.379
**TSH** (mIU/L)	2.87 (3.00)	2.12 (1.64)	0.043 *
**TG** (mg/dL)	138.32 (58.63)	93.1 (45.48)	0.000 *
**CHOLESTEROL** (mg/dL)	181.67 (40.20)	168.25 (29.29)	0.096
**LDL** (mg/dL)	103.37 (30.73)	97.2 (24.90)	0.336
**HDL** (mg/dL)	48.77 (9.86)	52.77 (13.38)	0.137
**CRP** (mg/dL)	9.45 (9.56)	3.45 (1.30)	0.001 *
**GLU** (mg/dL)	98.5 (14.48)	94.52 (11.23)	0.179
**HOMA-IR**	5.80	2.30	0.001 *

* p-value < 0.05 was considered significant. PCOS, polycystic ovary syndrome; FSH, follicle-stimulating hormone; LH, luteinizing hormone; E2, estradiol; TSH, thyroid-stimulating hormone; TG, triglycerides; LDL, low-density lipoprotein; HDL, high-density lipoprotein; CRP, C-reactive protein; GLU, fasting blood glucose. Data are presented as mean  ±  SD. HOMA-IR, Homeostatic Model Assessment for Insulin Resistance. * Values in bold in the *p*-value column are those found to be statistically significant. ** Negative correlation.

**Table 4:** The metabolic and hormonal features of the study subgroups.

		*C. TRACTOMATIS IgG*			*H. PYLORI IgG*		
		*Negative* (n = 36)	*Positive* (n = 4)	*p-Value*	*Negative* (n = 12)	*Positive* (n = 28)	*p-Value*
**PCOS**	**TSH** (mIU/L)	3	1.78	0.243	2.4	3	0.610
	**TG** (mg/dL)	141	85	0.041 *	143	131	0.313
	**CHOLESTEROL** (mg/dL)	186	144	0.008 *	193	177	0.358
	**LDL** (mg/dL)	105.7	81.6	0.067	108.2	101.2	0.631
	**HDL** (mg/dL)	49	45	0.557	50	48	0.782
	**CRP** (mg/dL)	8.94	13.92	0.419	4	11.77	0.122
	**GLU** (mg/dL)	99	98	0.845	97	99	0.805
	**BMI** (kg/m^2^)	27.51	27.42	0.811	25.4	28.3	0.056
	**HOMA-IR**	8.1	3.5	0.528	6.7	8	0.805
		***Negative*** **(n = 38)**	***Positive*** **(n = 2)**	***p-Value***	***Negative*** **(n = 20)**	***Positive*** **(n = 20)**	***p-Value***
**CONTROL**	**TSH** (mIU/L)	1.96	5.72	0.400	2.04	2.26	0.779
	**TG** (mg/dL)	90	135	0.327	86	99	0.879
	**CHOLESTEROL** (mg/dL)	170	152	0.356	168	169	0.607
	**LDL** (mg/dL)	98	82	0.297	94	100	0.224
	**HDL** (mg/dL)	54	43	0.219	57	50	0.061
	**CRP** (mg/dL)	3.4	3	0.780	3.31	3.58	0.813
	**GLU** (mg/dL)	95	97	0.369	94	96	0.738
	**BMI** (kg/m^2^)	21.75	21.74	0.919	21.51	21.48	0.811
	**HOMA-IR**	2.64	2.01	0.697	2.6	2.5	0.620

* p-value < 0.05 was considered significant.

## Data Availability

All data generated or analyzed during this study are included in this published article.
